# The Efficacy of Therapeutic Respiratory Muscle Training Interventions in People with Bronchiectasis: A Systematic Review and Meta-Analysis

**DOI:** 10.3390/jcm9010231

**Published:** 2020-01-15

**Authors:** Rocio Martín-Valero, Ana Maria Jimenez-Cebrian, Jose A Moral-Munoz, Maria de-la-Casa-Almeida, Manuel Rodriguez-Huguet, Maria Jesus Casuso-Holgado

**Affiliations:** 1 Department of Physiotherapy, Faculty of Health Sciences, University of Malaga, Arquitecto Francisco Peñalosa 3, Ampliación de Campus de Teatinos, 29071 Malaga, Spain; 2 Department Nursing and Podiatry, Faculty of Health Sciences, University of Malaga, Arquitecto Francisco Peñalosa 3, Ampliación de Campus de Teatinos, 29071 Malaga, Spain; amjimenezc@uma.es; 3 Department Nursing and Physiotherapy, Faculty of Nursing and Physiotherapy, University of Cadiz, Avenue Ana de Viya 52, 11009 Cadiz, Spain; joseantonio.moral@uca.es (J.A.M.-M.); manuel.rodriguez@uca.es (M.R.-H.); 4 Institute of Research and Innovation in Biomedical Sciences of the Province of Cadiz (INiBICA), University of Cadiz, 11009 Cadiz, Spain; 5 Department Physiotherapy, Faculty of Nursing, Physiotherapy and Podiatry, University of Seville, C/Avicena s/n, 41009 Seville, Spain; mcasa@us.es (M.d.-l.-C.-A.);; 6 La Línea de la Concepción Hospital, 11009 Cadiz, Spain

**Keywords:** bronchiectasis, respiratory muscle training, respiratory muscle strength, maximum inspiratory pressure, maximum expiratory pressure, meta-analysis

## Abstract

Background: Respiratory muscle dysfunction is an important health problem with high morbidity and mortality and associated costs in patients with bronchiectasis (BC). The aim of this study was to analyse the effects of therapeutic respiratory muscle training (RMT) interventions on improving sputum clearance, ventilator function, muscle strength and functional capacity in BC. Methods: Systematic review and meta-analysis were conducted following PRISMA guidelines. Two independent investigators searched using several electronic databases. The methodological quality of nine studies was assessed using the PEDro scale. Study selection/eligibility criteria: The following were included: randomised controlled trials, randomised crossover trials and pilot studies of patients with BC that used the intervention as RMT (inspiratory/expiratory) and evaluations of respiratory muscle strength (maximal expiratory pressure/maximal inspiratory pressure). This systematic review was registered in PROSPERO (CRD42017075101). Nine studies were included, five of which obtained an A recommendation grade, three with B, and one with C. Study quality was poor to good (mean PEDro Score of 6.375 out of 10). Studies had small sample sizes (8–98). Results show improvements on PImax in favour of therapeutic respiratory muscle training intervention (MD = 6.08; 95% CI = 1.38, 10.77; *p* < 0.01; *I*^2^ = 92%). However, high heterogeneity was identified on meta-analysis.

## 1. Introduction

Bronchiectasis (BC) is a chronic respiratory condition characterised by bronchial dilatation secondary to airway inflammation, infection and dysfunction of mucociliary clearance [1,2,3,4]. BC, permanent damage and widening of one or more of the large connecting bronchi (airways) may occur in nearly one third of individuals with chronic obstructive pulmonary disease (COPD) [1]. A variety of factors may affect the efficacy of inspiratory muscle training, including the degree of lung hyperinflation and severity of airway obstruction [5]. Both cause dyspnoea, which is a symptom of respiratory failure; inspiratory muscle weakness may lead to muscle load and capacity discordance. 

BC is heterogeneous in its clinical features, causes and outcomes [6]. Respiratory secretions in individuals with BC have poor transport properties, which manifest as reduced mucociliary transport, higher contact angle, dyspnoea and decreased exercise tolerance [6]. BC is an important health problem with high morbidity, mortality and associated costs [7]. International guidelines for managing BC include referral to pulmonary rehabilitation (PR) that improves exercise tolerance and quality of life [8,9]. The current evidence stresses the importance of the future development of process and performance metrics to monitor PR programs, to be able to start international benchmarking, and to provide recommendations for international standards based on evidence and best practice [10]. Previous to therapeutic respiratory muscle training (RMT) intervention, airway clearance techniques (ACTs) have been an important component in the management of patients with bronchiectasis [11]. Previous studies indicated the importance of mobilising secretions and facilitating effective expectoration in order to get improving airway clearance [11,12,13].

There are two distinct types of specific therapeutic RMT interventions, namely respiratory muscle strength (resistive/threshold) training (RMST) and respiratory muscle endurance (hyperpnoea) training (RMET), which have been established to improve the endurance performance of healthy individuals [14]. Various methods of therapeutic RMT interventions have been described. Firstly, RMST performed by breathing against an external inspiratory and/or expiratory load. This load consists either of a flow-dependent resistance or of a pressure threshold that needs to be overcome and sustained to generate flow. When a patient breathes against an external expiratory load, these devices are known as positive expiratory pressure trainers (PEP). Secondly, patients perform concurrent inspiratory and expiratory muscle training (CRMT). Third, patients clean bronchial by oscillatory or temporary PEP training devices. Finally, target flow and target pressure respiratory muscle training is where patients are trained to generate a target respiratory flow rate through a fixed resistance.

Previous studies have supported, with a B recommendation grade, offering the use of IMT (inspiratory muscle training) in conjunction with conventional PR to enhance the maintenance of the training effect [15]. IMT provides breathing training together with resistance loading produced by a valve and was regarded as a mixture of strength and endurance training [16]. Two smaller studies yielded consistent results supporting the use of either inspiratory or expiratory pressure threshold load breathing exercises to improve pulmonary muscle strength in people with BC [5,17]. However, there are several unanswered issues regarding intensity, frequency and duration of training, as well as the optimal strategy for maintenance of benefit for patients with BC [18]. There is still scarce literature regarding the benefits of physical training and long-term effects in BC [12,19,20,21]. Therefore, it is necessary to establish the most effective management strategies at the earliest stage possible.

The major purpose of this research is to detect recommendation grades regarding therapeutic RMT interventions in BC. This systematic review and meta-analysis examined the available evidence of different modalities of therapeutic RMT interventions that improve sputum clearance and respiratory muscle weakness in BC. Hence, the most suitable protocol to improve the respiratory muscle weakness and the respiratory function must be established.

## 2. Methods

### 2.1. Search Strategy

This systematic review was performed following the PRISMA guidelines (Preferred Reporting Items for Systematic Reviews and Meta-Analysis) [22]. The PRISMA Checklist is detailed in Appendix A. The review protocol was registered with an international registration database (PROSPERO, Registration Number: CRD42017075101). All analyses were performed on data of previously published studies, and thus no ethical approval and patient consent were required. 

A literature search was performed to identify the clinical studies that addressed the effect of instrumental mechanical devices for RMT in BC. The following databases were searched for relevant studies: MEDLINE (PubMed), Physiotherapy Evidence Database (PEDro), Cochrane Database of Systematic Reviews and CINAHL. Two reviewers carried out several searches in the databases using combinations of key words: bronchiectasis, respiratory muscle training, respiratory muscle strength, maximum inspiratory pressure and maximum expiratory pressure. The search strategy is detailed in Appendix B. The research was limited to studies published between 2004 and November 2019.

### 2.2. Inclusion and Exclusion Criteria

Our research question was established following from the PICO (population, intervention, control/comparison and outcomes) model. First, this review describes non-cystic fibrosis BC confirmed in adults by clinical history, such as coughing, shortness of breath and exertional dyspnoea, pulmonary function tests and high-resolution computed tomography (HRCT) [5,17]. It was necessary to have a clinically stable disease with no requirements of antibiotics in the four weeks prior to starting the study [12,20,23]. Clinical trials, randomised controlled trials and pilot studies that used therapeutic RMT interventions were included.

The articles that did not use threshold trainers or did a postural drainage were excluded [24,25,26]. Studies were excluded if steroids (inhalation or oral) such as Salbutamol were used [27]. Studies were excluded if patients were experiencing an acute exacerbation [25]. An acute exacerbation was defined as the presence of four out of 12 symptoms of a respiratory exacerbation, and requiring a course of oral antibiotics [28].

### 2.3. Quality Assessment and Data Extraction

Two independent reviewers (MJCH and RMV) completed the assessment list based on the PEDro score. This scale (0–10) is based on the list developed by Verhagen et al. [29], and assesses the internal validity of randomised controlled trials. A study with a PEDro score of 6 or more is considered level-1 evidence (6–8: good; 9–10: excellent) and a score of 5 or less is considered level-2 evidence (4–5: fair; <4: poor) [30]. The methodological quality of the eight studies was evaluated using the PEDro scale [30,31,32]. Disagreements between authors were initially resolved via discussion, and then by consultation with a third reviewer (JAMM).

The following characteristics were extracted: different therapeutic RMT interventions, namely inspiratory or expiratory exercises. Firstly, therapeutic inspiratory muscle intervention could be performed with different intensities and duration exercises through resistance offered by a valve threshold, adapting to the needs and changes of the individual as it progressed through the study. Secondly, therapeutic expiratory muscles intervention included four different types of instrumental mechanical devices: flutter, Acapella, UNIKO-TPEP^®^ and Origen-Dual Valve^®^.

The outcome measures included were maximum inspiratory pressure (PImax) [5,17], maximum expiratory pressure (PEmax) [5,12,20,23], respiratory muscle strength, forced vital capacity (FVC) [5,12,20,27], and maximum oxygen consumption or VO_2_ peak [17]. Other used measures included the sputum volume (SV) [11] or measurement of respiratory mechanics associated with peripheral airway resistance (such as the first derivative of resistance as a function of frequency) [33], transport velocity (TV) [34], exercise capacity (Six Minute Walk test (6MWT)) [5,20] and hand dynamometry (using a Jamar hydraulic) [21]. Regarding anthropometric outcomes related with body composition, Body Mass Index (BMI) and Fat Free Mass Index (FFMI) with Dual-energy X-Ray Absorpiometry (DEXA), mid-arm muscle circumference (MAMC), and phase angle by Bio-impedance were found [21]. Self-Reported outcomes measures included The Borg Scale, St George’s Respiratory Questionnaire [5,12,20], Quality of Life Questionnaire for Bronchiectasis (QOL-B-Spain-v3.0) and a seven-day prospective dietary questionnaire [21].

The results for the primary and secondary outcome measures were described and where possible study results were pooled and meta-analysis was conducted. Effect size (ES) was calculated using the difference between the mean (MD) and the standard deviation of the difference (SMD) with 95% confidence intervals (95% CI). An alpha value <0.05 was considered as statistically significant. We decided to pool studies based on comparing the RMT and control group.

The ES values were used to compare the different modalities of therapeutic RMT intervention with control group. Analysis of the effect size values was based on Cohen’s work, which determined that values below 0.2 were considered to have no effect, those between 0.2 and 0.5 as small effect, those between 0.5 and 0.8 a medium effect, and those above 0.8 a major effect [35]. The recommendation grades were studied according to the Duodecim (Finnish Medical Society Duodecim), a clinical practice guide developed in Finland to improve the quality of health care [36]. Grade A means that the recommendation is based on strong evidence. Grade B is based on sufficient evidence to make a clear recommendation. Grade C recommendations are based on limited evidence. Grade D refers to recommendations for which there is no evidence based on clinical studies [37].

### 2.4. Statistical Analysis

A meta-analysis was applied to compare changes in the effect size (post-intervention and pre-intervention) between the intervention group and the control group. For the meta-analysis, the standardised mean difference was calculated along with the 95% confidence interval, with a significance level set to *p* < 0.05. Heterogeneity was determined by the chi-square test and the *I*^2^ statistic. The results of all the subgroups included in this meta-analysis were represented in Forest plots. The statistical analyses were carried out with the statistical software REVIEW MANAGER 5.3 (The Cochrane Collaboration) (The Nordic Cochrane Centre 2014).

## 3. Results

### 3.1. Study Selection and Methodological Quality Assessment

**Figure 1:**
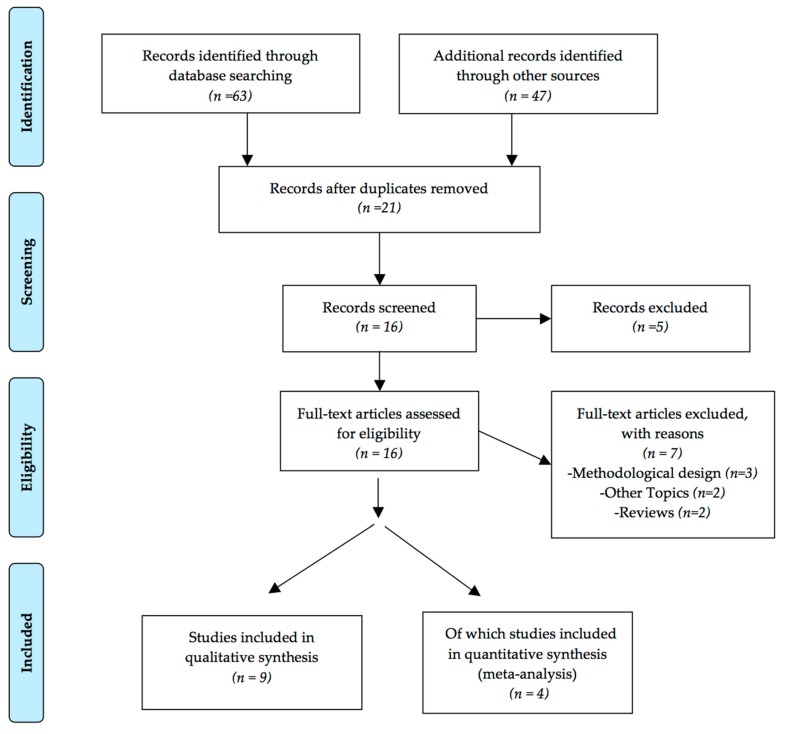
PRISMA flow chart of the study screening and selection process.

The initial database searches returned sixty-three potential studies. Sixteen relevant papers were found in the search strategy, and nine studies fulfilled the inclusion criteria (Figure 1). After reviewing titles and abstracts, fifteen papers were selected for full-text evaluation. Of these, nine papers were included in the systematic review and meta-analysis. Table 1 shows the assessment of methodological quality according to PEDro scale (mean PEDro score of 6.375 out of 10). We found seven studies [5,17,21,23,33,38,39] with level 1 evidence (good; 75% [7/9]); one study [20] had score of 5, which is considered level 2 evidence (acceptable; 12.5% [1/9]); and another study [12] had scores of 4 or less, which is considered (poor; 12.5% [1/9]). Trials were considered of enough methodological quality if they had a score of at least 5 out of 10 points. This was based on the fact that the tests with a score close to 4 do not employ a triple blind methodology (i.e., patient, evaluator and providing treatment).

### 3.2. Study Design and Population Characteristics

Nine studies with a total of 262 participants clinically diagnosed with BC were included in this systematic review. Protocol characteristics, intensity of training, recommendation grades, effect size of clinical and self-reported outcomes are described in Table 2. Regarding therapeutic RMT interventions, namely inspiratory or expiratory exercises, we first found three studies: two randomised controlled trials [5,17] about therapeutic inspiratory muscle intervention. Besides, two different types of interventions were carried out [5,17]. Two groups were made: maximum controlled inspiratory training and non-intervention [5], in front of three groups were made: PR plus sham IMT (PR-SHAM), PR plus targeted IMT (PR-IMT), or control [17].

It was found that two of the studies reviewed used the Threshold trainer [5,17]. Regarding airway clearance, one study used Flutter VRP1 [40], one used Flutter valve TM [33], one used the UNIKO-TPEP^®^ [23], and two studies used oscillatory positive expiratory pressure (OPEP) device Acapella [12,20]. Furthermore, there is the Origen-Dual Valve [21], which allows both simultaneous and sequential dual training work (expiratory and inspiratory muscles).

### 3.3. Meta-Analysis

**Figure 2:**
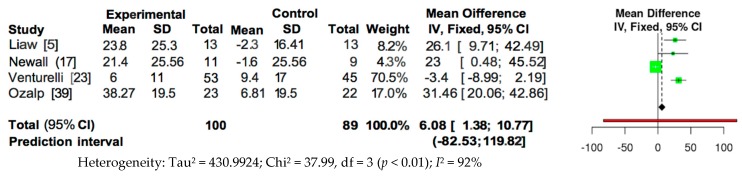
Forest plot for overall studies about PImax.

**Figure 3:**
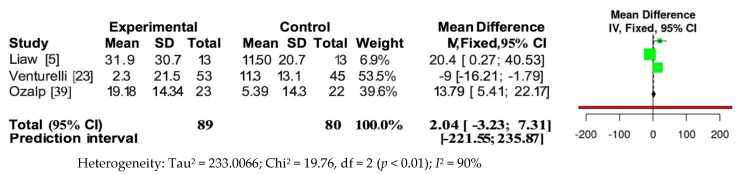
Forest plot for overall studies about PEmax.

The meta-analysis of the data of the RCTs was performed using the fixed effects model, as presented in Figure 2 and Figure 3. Because of the high heterogeneity, the sensitivity analysis was performed in relation to the type of training and intensity, analysing those RCT papers that used outcomes of PImax and PEmax. Therefore, the present review strengthens the evidence regarding the efficacy of respiratory muscle training for increasing respiratory muscle strength, because this review was based on meta-analyses of randomised trials with reasonable quality (mean PEDro Score of 6.375 out of 10).

Pooled analysis of the four studies measuring PImax showed a significant overall effect in favour of intervention (MD = 6.08; 95% CI = 1.38, 10.77; *p* < 0.01; *I*^2^ = 92%) (Figure 2). However, pooled analysis of the three studies measuring PEmax did not show a significant overall effect (MD = 2.04; 95% CI = −3.33, 7.31; *p* < 0.01; *I*^2^ = 90%) (Figure 3).

## 4. Discussion

This systematic review summarises recommendation grades of various therapeutic RMT interventions in BC. It was observed that RMT may improve PImax and PEmax strength of respiratory muscles in this population [5,17,23]. These benefits in respiratory muscles have also been found in patients with multiple sclerosis (MS) [41] and chronic obstructive pulmonary disease (COPD) [42]. There are scarce studies about RMT in BC. After performing this systematic review, eight studies about therapeutic RMT intervention in BC were carried out [5,12,17,20,21,23,33,40]. Three studies reviewed used the Threshold trainer [5,17]. Three studies were conducted about bronchial clearance with PEP with Flutter VRP1 [33,40], three studies used Acapella [12,20], one used Origen-Dual Valve^®^ [21], and one used the UNIKO-TPEP^®^ [23]. RMT could be performed using either inspiratory or expiratory muscle devices. Only one study compared the value of inspiratory versus expiratory training in patients with BC [11].

Three primary documents with an “A” recommendation grade for IMT were included in this review [5,17,39]. According to the treatment with positive expiratory pressure (PEP), two studies were found with an “A” recommendation grade where there were improvements on lung function and symptoms in patients with chronic lung disease and mucus hyper-secretion [12,23].

Firstly, Newall et al. carried out an IMT protocol of eight weeks, three days per week, two sets per day and 15 min per set, with an intensity of 30% MIP with Threshold Trainer, which was increased by 5% each week until a training intensity of 60% PImax was reached [17]. Three groups were made: a PR plus sham IMT (PR-SHAM), a PR plus targeted IMT (PR-IMT), and a control group, which did not carry out the training. At the end of the protocol, the PR-SHAM increased the PImax +12 cm H_2_O (1.1 to 22.9 cm H_2_O) and the PR-IMT increased the PImax +21.4 cm H_2_O (9.3 to 33.4). The PImax for the control group came down −1.6 (−6.2 to 3.0). Regarding exercise capacity, Newall et al. found improvement for the incremental shuttle walking test (ISWT) in the three groups (PR-SHAM +96.7 m, PR-IMT +124.5, control group +11 [17].

Secondly, Liaw et al. performed an IMT protocol of eight weeks of duration, for minimum five days per week, 30 min per day, starting with an intensity of 30% of PImax and increasing it 2 cm H_2_O each week. At the end of the protocol, all outcomes were statistically significant. Thus, the effect sizes increased for PImax + 23.85 cm H_2_O (*p* = 0.004), PEmax was + 31.92 cm H_2_O (*p* = 0.004), the 6MWT + 61.31 (*p* = 0.021) and the FVC was + 2.51% predictive (pred) (*p* = 0.309). Other outcomes such as the Borg Scale and the St George’s Respiratory Questionnaire also improved, coming down 1.46 and 32.46, respectively [5]. However, only one study measured with Leicester Cough Questionnaire [39].

Third, Venturelli et al. also carried out an EMT protocol of 10 days, twice a day, for 15 min with the Positive Expiratory Pressure (PEP) divide plus 20-min cycles of manually assisted breathing techniques. At the end of the protocol, PImax was increased + 6% pred (*p* = 0.541), PE max increased + 2.3% pred (*p* = 0.233) and the FVC increased + 4.3% pred (*p* = 0.495) [23]. The treatment with the PEP showed improvements in PImax and PEmax, with an increase of 6% pred and 2.3% pred, respectively. Therefore, temporary PEP not only improves symptoms in patients with chronic pulmonary disease and mucus hypersecretion, but also improves the strength and endurance of respiratory muscles [23].

Murray et al. performed an EMT protocol with Acapella for 12 weeks, twice daily, for 20–30 min per session, and three sets of 10 breaths per set. Thus, the effect sizes increased for PImax + 4.5 cm H_2_O (*p* = 0.2), the FVC was + 0.18 L (*p* = 0.6), but came down for PEmax −1.5 cm H_2_O (*p* = 0.3). At the end of the protocol, only St George’s Respiratory Questionnaire was statistically significant, coming down 0.7 (*p* = 0.004) [12]. This study found that regular chest physiotherapy in BC has significant benefits compared with no chest physiotherapy [12].

Moreover, one pilot study with “C” recommendation grade used Acapella for eight weeks [20]. Mandal et al. found improvements of respiratory muscle strength after therapeutic RMT intervention, where a chest physiotherapy group and chest physiotherapy plus pulmonary rehabilitation group were performed [20]. Both groups improved their outcomes, but the improvements in the chest physiotherapy plus pulmonary rehabilitation group (IG) (PImax + 6.6 cm H_2_O, PEmax + 14.7 cm H_2_O, FVC 0.2L) were greater than those of the chest physiotherapy group (CG) (PImax + 5.9 cm H_2_O, PEmax + 5.3 cm H_2_O, FVC 0L). Regarding physical capacity, the incremental shuttle walking test also increased in the chest physiotherapy plus pulmonary rehabilitation (+56.7 m) and the St George’s Respiratory Questionnaire also improved, decreasing 8 in IG and 1.4 in CG [20]. Furthermore, regarding airway clearance devices, chest physiotherapy with Acapella should be carried out for 12 [12] or 8 weeks [20], twice a day according to revised studies in this systematic review.

Only one study evaluated the effect of PR versus PR plus hyperproteic oral nutritional supplement enriched with beta-hydroxy-beta-methylbutyrate (HMB) on body composition, health related quality of life, skeletal muscle strength and plasma levels of prealbumin and myostatin. People with BC performed PR coupled with 15 min of breathing retraining with the Orygen-Dual Valve^®^, which allows both simultaneous and sequential dual training work (expiratory and inspiratory muscles) [21].

## 5. Limitations

There were two limitations on therapeutic RMT interventions for people with BC. First, small sample sizes reduced the ability to detect the effects of treatment. In addition, studies were designed with a follow-up period that was not long enough, with 12 weeks as the longest protocol [21]. Therefore, it might be interesting to extend the intervention period to six months or even a year.

## 6. Future Research

Further research would be necessary to consider the effects that different training protocols (duration of the inspiratory or expiratory training, frequency of sessions, high intensity of the respiratory muscles training program, and exercise capacity) may have on people with BC and to determine the range of the changes in outcomes associated with respiratory muscles training. It would also be necessary to take into account a training protocol for the respiratory muscles proposed, in which inspiratory muscle training, expiratory muscle training or both types of respiratory training work together for people with BC.

## 7. Clinical Implications

To our knowledge, this is the first systematic review to include only randomised or quasi-randomised clinical trials and to examine the effects of respiratory muscle training on the inspiratory and expiratory muscles for people with BC.

## 8. Conclusions

There were improvements in the strength of the respiratory muscles during therapeutic RMT intervention with a threshold trainer as a way of treatment for muscles weakness. Three revised articles with “A” grade recommendation covered a protocol of eight weeks, with a frequency of three or five days per week, with one or two daily sessions, each session consisting of 30 min per day or 15 min per set, two sets per day, with an intensity of 30% of PImax.

## Figures and Tables

**Table 1:** PEDro score for Methodological Quality assessment of nine studies.

Section/Theme	Ozalp [39]	Venture Lli [23]	Liaw [5]	Newall [17]	Olveira [21]	Tambascio [40]	Figueiredo [33]	Mandal [20]	Murray [12]
Eligibility criteria	Yes	Yes	Yes	Yes	Yes	Yes	Yes	Yes	Yes
Randomly allocated	Yes	Yes	Yes	Yes	Yes	Yes	Yes	Yes	Yes
Concealed allocation	No	Yes	Yes	Yes	Yes	Yes	Yes	No	No
Comparability of base	Yes	Yes	Yes	Yes	Yes	Yes	No	Yes	No
Blinding of subjects	Yes	Yes	No	No	No	No	No	No	No
Blinding of therapist	No	No	Yes	No	No	No	Yes	No	No
Blinding of assessor	Yes	No	Yes	No	No	No	No	No	No
Proper Continuation	Yes	Yes	No	Yes	Yes	No	No	Yes	Yes
Intention to treat	Yes	Yes	Yes	Yes	Yes	Yes	Yes	No	No
Between-group statistical comparison	Yes	Yes	Yes	Yes	Yes	Yes	Yes	Yes	Yes
Point measure and measures of variability	Yes	Yes	Yes	Yes	Yes	Yes	Yes	Yes	Yes
Total	**8**	**8**	**8**	**7**	**7**	**6**	**6**	**5**	**4**

**Table 2:** Key findings of primary studies.

Study, DR	PEDro, Type of Study, Sample Size	Training Protocol	Intra-Group Outcomes	Effect Size (%) Clinical OC Measures	Effect Size (%) Self-Reported OC Measures
Venturelli, 2013 [23] A	PEDro: 8/10 Randomised controlled trial *n* = 98	10 days twice a day 20-min cycles of manually assisted breathing techniques plus 15 min of temporary PEP	PImax *p* = 0.541 PEmax *p* = 0.233 FVC *p* = 0.495	PImax + 6% pred PEmax + 2.3% pred FVC + 4.3% pred	
Liaw, 2011 [5] A	PEDro: 8/10 Randomised controlled trial *n* = 26	8 week 5 days/week 30 min/day I: 30% MIP increasing 2 cm H_2_O/week	**↑**PImax *p* = 0.004 **↑**PEmax *p* = 0.004 **↑**6MWD *p* = 0.021 FVC *p* = 0.309	PImax + 23.85 cm H_2_O PEmax + 31.92 cm H_2_O 6MWD + 61.31 m FVC + 2.51% pred	TBS 1.46 SGRQ 32.46
Ozalp, 2019 [39] A	PEDro: 8/10 Randomised controlled trial *n* = 45	8 week 3 days/week Only 1 ss/week was performed under the supervision I: target workload was selected from 30% (first session) to 70% (third session) of MIP	**↑**PImax *p* = 0.001 **↑**PEmax *p* = 0.233 SIP *p* < 0.05 ISWT distance	PImax + 2.62 cm H_2_O PEmax + 1.039 cm H_2_O	**↓**FSS −0.197 *p* = 0.05 **↑**LCQ 0.52 *p* = 0.05
Newall, 2005 [17] A	PEDro: 7/10 Randomised controlled trial *n* = 32	8 week 3 days/week 2 sets/day 15 min/set I: 30% MIP and increased 5% each week until a training I of 60% MIP	**↑**PImax *p* = 0.003 Vo_2_ peak *p* = 0.192	PImax (cm H_2_O): PR-sham 12, PR-IMT 21.4; PR-control − 1.6 Vo_2_ peak(ml/min/kg): PR-sham 1.96; PR-IMT 0.35; PR-control − 1.91 ISWT (m): PR-sham 96.7; PR-IMT 124.5 PR-control 11 EE (m): PR-sham 392.8 PR-IMT 607.3; PR-control − 112.6	
Murray, 2009 [12] A	PEDro: 4/10 Randomised crossover trial *n* = 20	3 months of twice daily, each ss 20–30 min, 3 sets, 10 breath/set	PImax *p* = 0.2 PEmax *p* = 0.3 FVC *p* = 0.6	PImax +4.5 cm H_2_O PEmax − 1.5 cm H_2_O FVC + 0.18 L	**↓** SGRQ 0.7 *p* = 0.004
Mandal, 2012 [20] C	PEDro: 5/10 RCT *n* = 30	8 weeks, 3sets/ss, 20–30 min/ss Twice a day CG: chest physiotherapy IG: chest physiotherapy plus PR		CG: PImax + 5.9 cm H_2_O PEmax + 5.3 cm H_2_O ISWT − 4.6 m IG: PImax + 6.6 cm H_2_O PEmax + 14.7 cm H_2_O ISWT + 56.7 m	CG: SGRQ 1.4 IG: **↓** SGRQ 4 *p* < 0.001
Tambascio, 2011 [40] B	PEDro: 6/10 RCT crossover *n* = 18	4 weeks with Flutter VRP1^®^ 30 min daily and 1 weeek of a “wash-out” period vs. Flutter therapy		Flutter Therapy **↓**CAM: 6.11 ± 0.5° *p* > 0.05	
Figueiredo, 2012 [33] B	PEDro: 6/10 RCT crossover *n* = 8	Flutter Valve TM vs Sham Flutter (placebo)		8.4 mL more secretions	
Olveira, 2015 [21] B	PEDro: 7/10 RCT parallel groups *n* = 30	12 weeks PR 45 min +15 min with Orygen-Dual Valve^®^ 3 days/week (one unsupervised ss)		BMD: 0.013 ± 0.002 FFM: 1.1 ± 0.1 *p* > 0.01 **↑**FFMI:0.4 *p* > 0.01 Maximal Handgrip: 2.2 *p* > 0.01	QOL-B: 8.1 ± 4.6 *p* > 0.05

## Abbreviations

BMD	bone mineral density
CAM	contact angle measurement
CG	control group
DR	Degrees of Recommendation
EE	endurance exercise
FFM	Fat free mass
FFMI	Fat free mass index
FVC	forced vital capacity
FRC	functional residual capacity
FSS	Fatigue Severity Scale
I	intensity
IG	intervention group
ISWT	incremental shuttle walking test
LCQ	Leicester Cough Questionnaire
MBC	maximum breathing capacity
PEmax	maximal expiratory pressure
MIE	maximal inspiratory effort
min	minute
PImax	maximal inspiratory pressure
OC	outcome
OPEP	oscillating positive expiratory pressure
PEDro	Physiotherapy Evidence Database
PEP	positive expiratory pressure
PPS	patient preference scale
PRISMA	Preferred Reporting Items for Systematic Reviews and Meta-Analysis
PR	pulmonary rehabilitation
PR-control	pulmonary rehabilitation control group
PR-IMT	pulmonary rehabilitation inspiratory muscle training group
PR-sham	pulmonary rehabilitation sham group
QOL-B	quality of Life for BC
SGRQ	St George’s Respiratory Questionnaire
RV	residual volume
ss	session
SIP	sustainable inspiratory pressure
SV	sputum volume
TBS	The Borg Scale
TIMT	threshold inspiratory muscle trainer
TLC	Total Lung Capacity
TV	transport velocity
VRP1^®^	type of Flutter
6MWD	6 Minute-Walking Distance

## Appendix A. PRISMA 2009 Checklist

**Table:**

**Title**			
Title	1	Identify the report as a systematic review, meta-analysis, or both.	1
**Abstract**			
Structured summary	2	Provide a structured summary including, as applicable: background; objectives; data sources; study eligibility criteria, participants, and interventions; study appraisal and synthesis methods; results; limitations; conclusions and implications of key findings; systematic review registration number.	Abstract page
**Introduction**			
Rationale	3	Describe the rationale for the review in the context of what is already known.	1–2
Objectives	4	Provide an explicit statement of questions being addressed with reference to participants, interventions, comparisons, outcomes, and study design (PICOS).	3
**Methods**			
Protocol and registration	5	Indicate if a review protocol exists, if and where it can be accessed (e.g., Web address), and, if available, provide registration information including registration number.	3
Eligibility criteria	6	Specify study characteristics (e.g., PICOS, length of follow-up) and report characteristics (e.g., years considered, language, publication status) used as criteria for eligibility, giving rationale.	3–4
Information sources	7	Describe all information sources (e.g., databases with dates of coverage, contact with study authors to identify additional studies) in the search and date last searched.	3
Search	8	Present full electronic search strategy for at least one database, including any limits used, such that it could be repeated.	Appendix B
Study selection	9	State the process for selecting studies (i.e., screening, eligibility, included in systematic review, and, if applicable, included in the meta-analysis).	3–4
Data collection process	10	Describe method of data extraction from reports (e.g., piloted forms, independently, in duplicate) and any processes for obtaining and confirming data from investigators.	3
Data items	11	List and define all variables for which data were sought (e.g., PICOS, funding sources) and any assumptions and simplifications made.	4
Risk of bias in individual studies	12	Describe methods used for assessing risk of bias of individual studies (including specification of whether this was done at the study or outcome level), and how this information is to be used in any data synthesis.	5–11
Summary measures	13	State the principal summary measures (e.g., risk ratio, difference in means).	5–11
Synthesis of results	14	Describe the methods of handling data and combining results of studies, if done, including measures of consistency (e.g., I^2^) for each meta-analysis.	4

**Table:**

**Section/Topic**	**#**	**Checklist Item**	**Reported on Page #**
Risk of bias across studies	15	Specify any assessment of risk of bias that may affect the cumulative evidence (e.g., publication bias, selective reporting within studies).	4
Additional analyses	16	Describe methods of additional analyses (e.g., sensitivity or subgroup analyses, meta-regression), if done, indicating which were pre-specified.	Not aplicable
**Results**			
Study selection	17	Give numbers of studies screened, assessed for eligibility, and included in the review, with reasons for exclusions at each stage, ideally with a flow diagram.	Page 5, Figure 1
Study characteristics	18	For each study, present characteristics for which data were extracted (e.g., study size, PICOS, follow-up period) and provide the citations.	Page 7–9, Table 2
Risk of bias within studies	19	Present data on risk of bias of each study and, if available, any outcome level assessment (see item 12).	Table 1
Results of individual studies	20	For all outcomes considered (benefits or harms), present, for each study: (a) simple summary data for each intervention group (b) effect estimates and confidence intervals, ideally with a forest plot.	Table 2 Figure 2 and Figure 3
Synthesis of results	21	Present results of each meta-analysis done, including confidence intervals and measures of consistency.	Figure 2 and Figure 3
Risk of bias across studies	22	Present results of any assessment of risk of bias across studies (see Item 15).	Not aplicable
Additional analysis	23	Give results of additional analyses, if done (e.g., sensitivity or subgroup analyses, meta-regression [see Item 16]).	Not aplicable
**Discussion**			
Summary of evidence	24	Summarize the main findings including the strength of evidence for each main outcome; consider their relevance to key groups (e.g., healthcare providers, users, and policy makers).	9–11
Limitations	25	Discuss limitations at study and outcome level (e.g., risk of bias), and at review-level (e.g., incomplete retrieval of identified research, reporting bias).	13
Conclusions	26	Provide a general interpretation of the results in the context of other evidence, and implications for future research.	14
**Funding**			
Funding	27	Describe sources of funding for the systematic review and other support (e.g., supply of data); role of funders for the systematic review.	14

From: Moher D, Liberati A, Tetzlaff J, Altman DG, The PRISMA Group (2009). Preferred Reporting Items for Systematic Reviews and Meta-Analyses: The PRISMA Statement. PLoS Med 6(7): e1000097. doi:10.1371/journal.pmed1000097.

## Appendix B. Detailed Search Strategy

**N° Terms used**

#1 “Respiratory muscle training”

#2 “Respiratory muscle strength”

#3 “Maximum inspiratory pressure”

#4 “Maximum expiratory pressure”

**#5 “Bronchiectasis”**

**PUBMED (63 potential articles):**
((((((“respiratory muscle training” [Title/Abstract]) OR “respiratory muscle strength” [Title/Abstract]) OR “maximum inspiratory pressure” [Title/Abstract]) OR “maximum expiratory pressure”[Title/Abstract]) AND “bronchiectasis” [Title/Abstract] Filters: Clinical Trial; Randomized Controlled Trial.

**PEDro (3 potential articles):**

- #1 AND #2AND #5 (1 potential article AND 1 RS)
- #2 AND #5(1 potential article AND the same RS)
- #3 AND #5 (1 potential article AND the same RS)
- #4 AND #5(1 potential article)

**Cochrane Database of Systematic Reviews (4 potential articles):**

- #1 AND #2 AND #5 (1 potential articles)
- (#1 OR #2) AND #5(2 potential articles)
- (#3 OR #4) AND #5 (1 RS)
- #3 AND #4 AND #5 (2 potential articles)
- (#1 OR #2 OR #3) AND #5 (2 potential articles)
- (#1 OR #2 OR #4) AND #5 (2 potential articles)
- (#1 OR #3 OR #4) AND #5 (1 potential article AND 1 RS)
- (#2 OR #3 OR #4) AND #5 (1 potential article)

**CINAHL (40 potential articles):**

- #1 AND #5 (10 potential article)
- #5 AND (#1 OR #2) (30 potential articles)
- #3 AND #4 AND #5 (1 potential article)
- #3 AND #5 (1 potential article)
- #4 AND #5 (1 potential article)

**Note:** only search strategies with results are shown.
